# The Impact of the COVID-19 Pandemic on Youth with Chronic Pain and Their Parents: A Longitudinal Examination of Who Are Most at Risk

**DOI:** 10.3390/children9050745

**Published:** 2022-05-19

**Authors:** Kathryn A. Birnie, Daniel C. Kopala-Sibley, Maria Pavlova, Cara G. Nania, Emily Bernier, Jennifer N. Stinson, Melanie Noel

**Affiliations:** 1 Department of Anesthesiology, Perioperative, and Pain Medicine, Alberta Children’s Hospital Research Institute, University of Calgary, Calgary, AB T2N 4N1, Canada; 2 Department of Community Health Sciences, University of Calgary, Calgary, AB T2N 4N1, Canada; 3 Alberta Children’s Hospital Research Institute, University of Calgary, Calgary, AB T2N 1N4, Canada; daniel.kopalasibley@ucalgary.ca (D.C.K.-S.); melanie.noel@ucalgary.ca (M.N.); 4 Hotchkiss Brain Institute, University of Calgary, Calgary, AB T2N 1N4, Canada; 5 The Mathison Centre for Mental Health Research & Education, University of Calgary, Calgary, AB T2N 4Z6, Canada; 6 Department of Psychiatry, University of Calgary, Calgary, AB T2N 1N4, Canada; emily.bernier@ucalgary.ca; 7 Department of Psychology, University of Calgary, Calgary, AB T2N 1N4, Canada; mpavlova@ucalgary.ca (M.P.); cgnania@ucalgary.ca (C.G.N.); 8 Child Health Evaluative Sciences, Research Institute, The Hospital for Sick Children, Toronto, ON M5G 1X8, Canada; jennifer.stinson@sickkids.ca; 9 Lawrence S. Bloomberg Faculty of Nursing, University of Toronto, Toronto, ON M5T 1P8, Canada

**Keywords:** chronic pain, COVID-19 pandemic, youth, parents, mental health, personality

## Abstract

Objectives: Chronic pain and mental illness in youth and parents are poised to reach new heights amidst the societal and healthcare impacts of the COVID-19 pandemic. Evidence from natural disasters (i.e., hurricanes) suggests that a degree of personal impact and individual personality may moderate the effects of high stress events, such as the COVID-19 pandemic, on mental health. Methods: In a pre-existing cohort of 84 youth with chronic pain (*M*_age_ = 14.39; 12–18 years; 67.8% female) and 90 parents (86.7% female), we examined changes in youth pain interference and youth and parent mental health (depression, anxiety) from before to during the first wave of the COVID-19 pandemic, and the influence of personal impact of the pandemic (i.e., financial, familial, health, social, occupational, and educational domains) and individual personality (neuroticism, conscientiousness, extroversion). Results: Overall, youth reported significantly lower pain interference and anxiety as compared to pre-pandemic; however, those more personally impacted by the pandemic reported worsening pain interference and anxiety symptoms. Overall, parents reported greater depressive symptoms as compared to pre-pandemic; however, those more personally impacted by the pandemic reported increased anxiety symptoms. Personality traits (high neuroticism, and low conscientiousness and extroversion) predicted worsened pain and mental health, and exacerbated effects of COVID-19-related personal impact on youth and parent anxiety symptoms. Discussion: Identifying risk and resilience profiles in youth and parents at high risk for worsening pain and mental health may better inform matching interventions to individual need.

## 1. Introduction

Chronic pain and mental illness in youth and adults were public health emergencies prior to the COVID-19 pandemic, with both affecting one in five individuals with lifelong and intergenerational impact [1,2,3,4]. Now, because of the COVID-19 pandemic, social isolation, and economic recession, rates of mental illness are poised to reach catastrophic rates [5,6,7,8,9,10], and early research suggests worsening chronic pain [11,12,13,14,15,16,17]. 

Chronic pain and mental health are integrally connected [18], as are the experiences of youth and their parents [1,2,19]. Youth with, versus without, chronic pain and their parents experience elevated anxiety and depression [18,20]. A study conducted with youth with chronic pain and their parents in the United States during the first wave of the COVID-19 pandemic found that high symptom burden remained stable or improved for youth pain interference, as well as youth and parent symptoms of insomnia, depression, and anxiety [21]. While direct exposures to COVID-19 did not modify trajectories, youth pain interference and parent insomnia worsened for those experiencing high economic stress. Another study of young adults with chronic pain revealed stability of pain and depression, but increases in anxiety during the COVID-19 pandemic [22]. However, direct exposures to COVID-19 and economic stress are not the only potential sources of stress posed by the pandemic. Furthermore, not all individuals will be equally vulnerable to exacerbated pain or mental health in the context of high stress [23,24,25,26]. There is an urgent need to identify which individuals are most at risk for the onset and worsening of pain and mental health. One underexplored consideration is individual personality.

Research suggests some relation between personality and mental health in chronic pain [27]. Mounting evidence indicates that personality moderates the effects of high stress on risk for mental health disorders [28,29,30,31,32]. In the aftermath of natural disasters (e.g., Hurricane Sandy), disaster-related stress predicted increased symptoms of depression and anxiety in youth and parents. However, the effects of disaster-related stress were heightened in youth and parents with high neuroticism (i.e., emotional instability, proneness to negative emotions, and susceptibility to stress) and in parents with low extroversion (i.e., being gregarious, social, and prone to positive affect) [33,34]. Conscientiousness (i.e., being organized, detail-oriented, and self-disciplined) may also be relevant in the COVID-19 pandemic. More conscientious individuals may cope better with public health restrictions, providing a greater sense of control and wellbeing, and decreased risk for depression and anxiety [35,36,37,38].

In a cohort of youth with chronic pain and their parents, we examined: (1) changes in youth pain interference and youth and parent symptoms of anxiety and depression from before to during the COVID-19 pandemic; (2) the influence of youth and parent personality traits on change in youth pain interference and youth and parent symptoms of anxiety and depression, (3) the effect of the degree to which youth and parents reported being personally impacted by the COVID-19 pandemic (i.e., financial, familial, health, social, occupational, and educational domains) on symptom change from prior to during the COVID-19 pandemic, and (4) whether personality traits moderated (i.e., exacerbated or buffered) effects of COVID-19 impact on pain and mental health. We expected worsened pain and mental health symptoms from prior to during the pandemic for youth and parents. Moreover, we expected that youth and parents who were more personally impacted by the pandemic to show greater increases in symptoms from prior to during the pandemic. We additionally expected higher neuroticism, lower extroversion, and lower conscientiousness to predict worsening mental health symptoms during the pandemic. Finally, we expected that neuroticism, conscientiousness, and extroversion would moderate the effects of COVID-19 impact on symptom change such that that greater personal impact from the COVID-19 pandemic would predict increases in pain interference in youth and depression and anxiety symptoms in parents and youth more strongly in those with elevated neuroticism or lower extroversion or conscientiousness.

## 2. Materials and Methods

### 2.1. Study Design

Families were eligible to participate in the current study if they had previously participated in the Pain and Mental Health in Youth (PATH) cohort, a prospective study examining the pain and mental health of youth with chronic pain and their parents, between 2017 and March 2020 [39,40,41,42,43]. The dataset analyzed during the current study is not publicly available. Youth in the PATH cohort were recruited from three outpatient clinics (Headache, Abdominal Pain, and Complex Pain) of a tertiary level children’s hospital in Western Canada. Eligibility for participation in the PATH cohort included youth between 10–18 years of age who had been identified by a healthcare provider as having chronic pain (i.e., pain ≥ 3 months [44]) without an underlying disease (e.g., cancer) and a parent. Exclusion criteria for both youth and parents included being unable to read/speak English, a diagnosis of a neurodevelopmental disorder (e.g., intellectual disability, autism spectrum disorder), and/or a serious mental health disorder (e.g., schizophrenia, psychotic disorder). When the COVID-19 pandemic occurred, and to reduce potential sources of bias, youth and parents who had previously participated in the PATH cohort were invited to participate in an additional follow-up timepoint focused specifically on the impact of the COVID-19 pandemic, including quantitative surveys (reported herein) and a qualitative interview. Results from the qualitative interviews will be reported elsewhere [45]. Of the 199 youth and parents who were part of the PATH cohort, 84 youth and 91 parents completed measures during the COVID-19 pandemic. The dataset analyzed during the current study is not publicly available. See Table 1 for demographic characteristics of the sample.

### 2.2. Procedure

Provinces across Canada declared states of emergency regarding the COVID-19 pandemic between 12–22 March 2020. Once institutional research ethics board was approved for the current study, research staff contacted eligible families from the PATH cohort via telephone/email to provide more information about the current study, invite participation and obtain informed consent. Youth and parents who agreed to participate consented or assented using an online consent (or assent) form through Research Electronic Data Capture (REDCap), a secure online data collection tool [46]. All study measures were also completed via REDCap between June–September 2020 (during the first wave of the pandemic). The COVID-19 impact measure assessed experiences up until completion of the measure. Youth and parents from the same families were not both required to participate (i.e., youth and/or parents could participate independent of the other family member’s decision to participate). 

### 2.3. Measures

COVID-19-related personal impact (see Supplementary Table S1). Youth and parents completed the 26-item COVID-19 Impact Questionnaire (youth and parent version) which assesses financial, familial, health, social, occupational, and educational impacts of the pandemic. The measure is drawn from published literature on the impact of natural disasters on mental health [33,34] and adapted and expanded to the unique nature of the COVID-19 pandemic (e.g., mandated isolation, being close to someone who was hospitalized/died, job loss, etc.). Higher scores indicate greater COVID-19 impact. Internal consistency for this measure was adequate in youth (alpha = 0.77) and parents (alpha = 0.76).

#### 2.3.1. Pain Interference

Youth completed the four-item pain interference subscale of the PROMIS-25 as a measure of their pain interference. This scale has excellent measurement properties [47,48]. Raw total scores are converted into *t*-scores that were used for analyses. Internal consistency was good in youth pre- and during the pandemic (alphas = 0.83–0.84). 

#### 2.3.2. Depressive and Anxiety Symptoms

Youth completed the 47-item Revised Child Anxiety and Depression Scale (RCADS) [49]; total raw scores were converted into *t*-scores that were used for analyses. Parents completed the 14-item Hospital Anxiety and Depression Scale (HADS) [50]. Both the RCADS and the HADS have excellent measurement properties [49,51,52]. Internal consistency was good to excellent in youth and parents both pre- and during pandemic (all alphas = 0.88–0.97). 

#### 2.3.3. Personality Traits

Neuroticism, extroversion, and conscientiousness were measured in youth and parents via self-report using the well-validated 44-item Big Five Inventory [32,37,53,54]. The Big Five Inventory has excellent measurement properties in both youth and adult samples [32,55,56,57,58]. Internal consistency for all three traits was good in both youth and parents (all alphas = 0.78–0.82).

### 2.4. Data Analyses

Analyses consisted of paired samples *t*-tests and hierarchical multiple regressions. Analyses adjusted for gender and age (for youth). We tested 9 models for youth and 6 for parents, predicting change pre- to during pandemic in pain interference and depressive and anxiety symptoms. Hierarchical regressions included three blocks (block 1: demographics and baseline symptoms; block 2: personality and COVID-19 impact; and block 3: interaction between personality and COVID-19 impact). Significant interactions were tested using Preacher and Hayes’ PROCESS macro [59]. Bootstrapping using 10,000 samples was used. Interactions were interpreted by comparing simple slopes at high and low levels (±1 SD) of the personality variable. We report unstandardized b-weights and standardized betas for each predictor, 95% confidence intervals around effects, and *R*^2^ for the total model and each block as measures of effect size. Missing data were handled by prorating the total scores if less than 20% of responses were missing; if more than 20% of responses were missing, data were excluded from analyses. Listwise deletion was used. 

## 3. Results

### 3.1. Participants

A total of 84 youth and 90 parents completed measures prior to March 2020 (pre-pandemic) and between June–September 2020 (during the pandemic). Sample size for each analysis is reported.

### 3.2. Descriptive Statistics

Mean levels of each stressor in the COVID-19 impact measure are shown in Supplementary Table S1. Means and standard deviations of study variables for youth and parents both prior to and during the pandemic are shown in Table 2. 

#### 3.2.1. Changes in Pain and Mental Health from pre- to during the COVID-19 Pandemic

Overall, youth pain interference and anxiety symptoms decreased (Table 2). Youth depressive symptoms did not change significantly. In parents, depressive symptoms increased from pre- to during the pandemic (Table 2). Parent anxiety symptoms did not change significantly. 

Predicting youth pain interference and mental health during the COVID-19 pandemic from COVID-19 impact and personality vulnerability (Table 3).

Controlling for impact, youth gender and age did not predict change in any outcome. Across models, higher youth pain interference, and depressive and anxiety symptoms pre-pandemic, respectively, predicted higher levels of youth pain interference, and depressive and anxiety symptoms during the pandemic. 

#### 3.2.2. Pain Interference

Greater COVID-19 impact predicted increased pain interference. Higher extroversion predicted decreased pain interference. The interactions were not significant.

#### 3.2.3. Depression

There was no significant effect of COVID-19 impact. Higher neuroticism and lower conscientiousness and extroversion predicted increased depressive symptoms. The interactions were not significant.

#### 3.2.4. Anxiety

**Figure 1:**
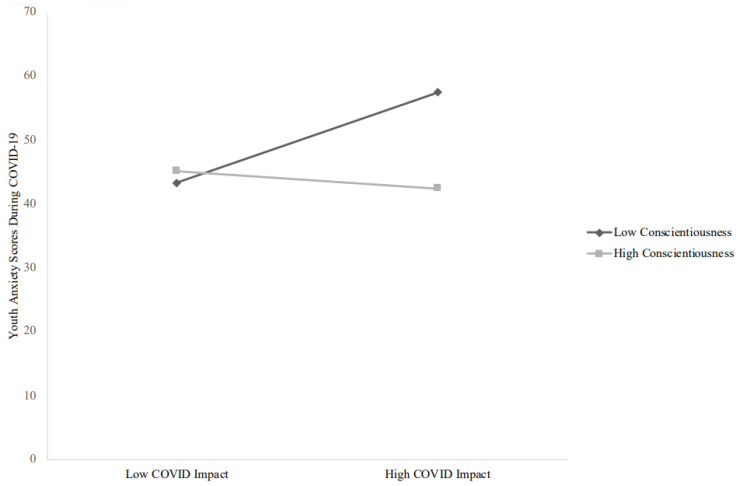
Interaction between COVID-19 impact and levels of conscientiousness on change in youth anxiety symptoms during COVID-19. Note: effects of COVID-19-related personal impact on mental health are depicted at ±1 standard deviation of the moderator.

Greater COVID-19 impact predicted increased anxiety symptoms, except when conscientiousness was controlled for. Higher neuroticism and lower conscientiousness and extroversion predicted increased anxiety symptoms. There was a significant interaction between COVID-19 impact and conscientiousness (Figure 1) such that greater impact predicted greater anxiety symptoms at low (*B* = 0.50, *t*(72) = 2.78, *p* = 0.007), but not high (*B* = −0.10, *t*(72) = −0.57, *p* = 0.57) levels of conscientiousness.

Predicting parent mental health during the COVID-19 pandemic from COVID-19 impact and personality vulnerability (Table 4).

Controlling for parent gender, COVID-19 impact, and baseline symptoms, across models, higher parent depressive and anxiety symptoms pre-pandemic, respectively, predicted higher parent depressive and anxiety symptoms during the pandemic. 

#### 3.2.5. Depression

COVID-19 impact did not predict change in depressive symptoms. Controlling for COVID-19 impact, higher neuroticism and lower extroversion predicted increased depressive symptoms. The interaction was not significant.

#### 3.2.6. Anxiety

**Figure 2:**
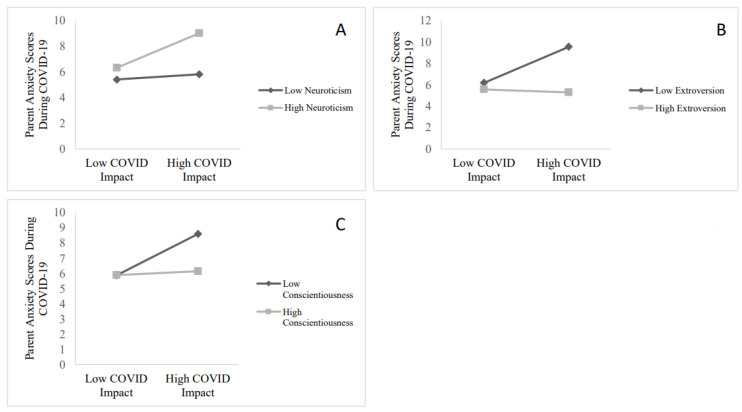
(**A**): Interaction between neuroticism and COVID-19 impact predicting change in parental anxiety symptoms. (**B**): Interaction between extroversion and COVID-19 impact predicting change in parental anxiety symptoms. (**C**): Interaction between conscientiousness and COVID-19 impact predicting change in parental anxiety symptoms. Note: effects of COVID-19-related personal impact on mental health are depicted at ±1 standard deviation of the moderator.

Greater COVID-19 impact predicted increased anxiety symptoms. Higher neuroticism and lower conscientiousness and extroversion predicted increased anxiety symptoms. There was a significant interaction between COVID-19 impact and neuroticism (Figure 2A), such that impact predicted increased anxiety symptoms at high (*B* = 0.10, *t*(87) = 3.55, *p* < 0.01), but not low, (*B* = 0.02, *t*(87) = 0.59, *p* = 0.59), levels of neuroticism. There was a significant interaction between COVID-19 impact and extroversion (Figure 2B) such that impact predicted increased anxiety symptoms at low (*B* = 0.13, *t*(87) = 4.13, *p* < 0.01), but not high (*B* = −0.01, *t*(87) = −0.36, *p* = 0.72) levels of extroversion. There was a significant interaction between COVID-19 impact and conscientiousness (Figure 2C) such that impact predicted increased anxiety symptoms at low (*B* = 0.10, *t*(87) = 3.03, *p* < 0.01), but not high (*B* = 0.01, *t*(87) = 0.34, *p* = 0.74), levels of conscientiousness. 

## 4. Discussion

An identified priority for COVID-19 health research is to inform responses to future infection waves or pandemics by identifying mechanisms to support prevention and treatment for vulnerable groups, such as those with pre-existing chronic pain and mental health issues [5,6,11,13,60,61]. This study addressed these priorities by examining how the COVID-19 pandemic has impacted the pain and mental health of youth with chronic pain and their parents. 

We expected worsened pain and mental health symptoms from prior to during the pandemic for youth and parents. Moreover, we expected that youth and parents who were more personally impacted by the pandemic to show greater increases in symptoms from prior to during the pandemic. We additionally expected higher neuroticism, lower extroversion, and lower conscientiousness to predict worsening mental health symptoms during the pandemic. Finally, we expected that neuroticism, conscientiousness, and extroversion would moderate the effects of the COVID-19 impact on symptom change such that that greater personal impact from the COVID-19 pandemic would predict increases in pain interference in youth and depression and anxiety symptoms in parents and youth more strongly in those with elevated neuroticism or lower extroversion or conscientiousness.

Our findings revealed that, on average, youth reported a decrease in pain interference and anxiety symptoms from before to during the COVID-19 pandemic, and parents experienced an increase in depressive symptoms. Youth and parents who had worse pain and mental health prior to the pandemic had worse pain and mental health during the pandemic. Youth who were more personally impacted by the pandemic experienced worsening pain interference and anxiety symptoms, while parents who were more personally impacted by the pandemic experienced worsening anxiety symptoms. This work extends prior examinations of the impact of the COVID-19 pandemic on pain and mental health in youth with chronic pain and their parents [21]. Specifically, we utilized a broader assessment of COVID-19 personal impact from the perspective of both youth and parents and examined the moderating role of personality traits. 

Consistent with prior research [27,28,29,30,33,34], youth and parents with higher neuroticism or lower extroversion or conscientiousness generally had worsening mental health. Furthermore, personality was found to buffer the personal impact of the COVID-19 pandemic on several domains of mental health for both youth and parents, but with no impact on youth pain interference. For youth, greater pandemic impact led to worsening anxiety, but only for those who were low in conscientiousness. For parents, greater personal impact of the pandemic led to worsening anxiety only for parents who were high in neuroticism or low in conscientiousness or extroversion. There were no interactions between COVID-19 personal impact and personality on youth pain interference or youth and parent depressive symptoms. Our findings of a moderating role of personality extends previous work showing the buffering role of low neuroticism and high extroversion in the relationship between the impact of natural disasters (i.e., Hurricane Sandy) on mental health in youth and parents [33,34]. Our work further extends this previous research by showing that low conscientiousness exacerbates the impact of the COVID-19 pandemic on subsequent anxiety symptoms in youth with chronic pain and their parents. 

While the mechanisms underpinning these interactions cannot be gleaned from the current study, individuals high on neuroticism may view negative events as more stressful or more hopeless than they actually are [62], employ less problem-focused and more avoidant coping strategies [36,63], and be less likely to seek out or receive instrumental social support [64], all of which may ultimately result in worsening mental health symptoms in the face of greater personal impact during the COVID-19 pandemic. Individuals high in extroversion may benefit from a greater ability to make decisions and plans under stressful situations given their greater cognitive resources [63]. They may also view difficult situations more optimistically and be more likely to solicit more instrumental social support [62,65]. Broaden-and-build models of positive affect suggest that the elevated levels of positive affect characteristic of individuals high in extroversion may facilitate thinking of novel and adaptive ways to cope with stress [66]. More conscientious individuals may be better able to reorganize their lives and structure their time following the upheaval from lock downs, social distancing, and remote schooling, to lessen the impact of the pandemic on their levels of anxiety symptoms. Another possibility is that more conscientious individuals were more observant of pandemic-related restrictions, such as social distancing and mask-wearing, thereby reducing their anxiety about contracting the virus by instilling them with a greater degree of perceived control. 

Consistent with some prior research with youth and adults with chronic pain [14,21], pain interference and mental health in youth with chronic pain in this study did not worsen overall in the first wave of the COVID-19 pandemic. This could be due to a decrease in the physical requirements of daily functioning that may be pain and/or anxiety provoking, albeit necessary to eventually reduce pain interference (e.g., physically attending school or activities). Nevertheless, while on average pain interference decreased, those who were more personally impacted by the pandemic (i.e., across financial, familial, health, social, occupational, and educational domains) reported increased pain interference from before to during the pandemic. This speaks to the differential impact of the COVID-19 pandemic between individuals and families. Greater negative impact of the pandemic has been identified in previous studies with adults with chronic pain related to reduced access to care, ability to self-manage pain, less personal resilience, and race (i.e., individuals identifying as Black and of non-Hispanic origin) [12,13,14,67]. The significant worsened outcomes for youth and parents with greater COVID-19 impact differs from the study by Law et al. [21] who found relatively minimal impact of direct COVID-19 exposure and financial impact on youth and parent mental health, suggesting the relevance of conceptualizing COVID-19 impact more broadly during this pandemic.

While overall pain interference and mental health for youth was not negatively affected, parental depressive symptoms worsened over time. Furthermore, personality and COVID-19 personal impact influenced changes in anxiety symptoms of youth with chronic pain and their parents. Emerging evidence points to the powerful influence of parental mental health on child chronic pain outcomes [1,2,68], suggesting potential risk posed to these youth in future. The cumulative impact of a prolonged stressor, such as the continued emergent waves of COVID-19 infections and accompanying restrictions, on pain and mental health in these families is unknown. 

These findings may have important clinical implications. Access to adequate mental health care in general, and to chronic pain care in particular, has been detrimentally impacted by the pandemic [12,14,69] with a rapid pivot to virtual delivery [70,71]. Identifying risk profiles in vulnerable individuals is critically needed to inform pain and mental health resource allocation to ensure youth access evidence-based care tailored to their individual needs. Furthermore, it is clear that interventions must address both pain and co-occurring mental health concerns [72]. Stepped care models that adjust levels of pain and mental health treatment to individual symptomatology and risk profiles have been recommended, including during the COVID-19 pandemic and for chronic pain [60,71,73,74,75]. It is possible that assessing personality traits and COVID-19-related personal impact, each of which were found to exacerbate risk for either worsening pain or mental health in youth and parents, may be useful to inform triage care and level intervention most appropriate for each family. Given the large percentage of symptom variance explained by our models (i.e., *R*^2^, see Table 3 and Table 4), our findings suggest that assessment of pre-COVID symptomatology, COVID-19-related personal impact, and personality traits may be highly useful in such treatment approaches.

Several limitations should be noted. First, assessment of pain, COVID-19-related personal impact, and mental health symptoms during the pandemic was completed during the first wave of the pandemic in Western Canada. Results may thus not generalize to subsequent infection waves. Second, pain, mental health symptoms, pandemic-related personal impact, and personality traits were assessed at the same time. It is thus possible that individuals with greater pain interference and mental health symptoms were more likely to report greater pandemic-related personal impact (i.e., recall bias). Further, participants were recruited from one major city in Western Canada; therefore, results may not generalize to other geographical regions that may impose different restrictions and have differing effects on the health care system, individuals, and society. The majority of the current sample was White, educated, and had medium to high socio-economic status, although household income spanned a relatively wide range. Research with more diverse populations is critically needed especially in light of the disproportionate impact of the COVID-19 pandemic on members of racial and ethnic minority groups, including those with chronic pain [67]. Finally, all measures were self-report and mono-informant, which may artificially inflate associations between personality, pandemic-related personal impact, and psychopathology. However, these limitations are less likely to account for the significant interactions found. 

Now well into the fourth wave, the COVID-19 pandemic may lead to a proliferation of pain and mental health problems in youth with chronic pain and their parents. Findings from this study suggest that the mental health impact of the COVID-19 pandemic in the first wave is not uniform but rather is influenced by the degree to which individuals are personally impacted as well as by their individual personality traits. By identifying individual risk profiles for worsening pain and mental health, we have the potential to ensure implementation of suited evidence-based interventions to reduce the long-lasting impacts of the COVID-19 pandemic across generations. 

## Figures and Tables

**Table 1:** Sociodemographic characteristics.

	(*n =* 84 Youth; *n* = 90 Parents)
Youth age (*M* years, *SD*)	14.39 (2.21)
Youth gender (% female)	67.8
Parent gender (% female)	86.7
Ethnicity	
White	83.5
Two or more ethnicities	8.8
Other	7.7
Aboriginal	4.4
Latin American	2.2
Black	2.2
Chinese	2.2
South-East Asian	2.2
Filipino	1.1
Arab/West Asian	1.1
Did not want to answer	2.2
Annual household income (%)	
<$10,000–$29,999	3.3
$30,000–$59,999	6.7
$60,000–$89,999	15.6
>$90,000	62.2
Did not want to answer	12.2
Parent education level (%)	
High school or less	7.8
Vocational school/some college	20.0
College, bachelors degree	52.2
Graduate/professional school	20.0

**Table 2:** Descriptive statistics and change in symptoms from prior to during the COVID-19 pandemic.

	Pre-COVID		During COVID							
	*n*	*M*	*n*	*M*	Mean Difference	SD	95% CI	*t*	*df*	*p*
Parents										
Depression	88	3.28	88	4.22	−0.93	2.79	−1.52 to −0.34	−3.13	87	<0.01 **
Anxiety	88	6.42	88	6.65	−0.23	3.05	−0.87 to 0.42	−0.70	87	0.486
COVID-19 impact			90	50.49		13.57				
Neuroticism			89	21.30		6.27				
Extroversion			89	31.53		6.38				
Conscientiousness			89	35.48		5.30				
Youth										
Pain interference	81	55.27	81	52.10	3.17	9.37	1.10 to 5.24	3.05	80	<0.01 **
Depression	80	55.26	80	54.92	0.34	14.54	−2.89 to 3.58	0.21	80	0.833
Anxiety	72	51.17	72	47.54	3.63	14.21	0.29 to 6.97	2.17	71	0.034 *
COVID-19 impact			84	51.90		13.49				
Neuroticism			84	25.25		6.76				
Extroversion			84	27.78		6.72				
Conscientiousness			84	30.08		6.42				

Abbreviation: *n*, sample size; *M*, sample mean; SD, standard deviation; CI, confidence interval, *t*, *t*-test value, *df*, degrees of freedom. * *p* < 0.05, ** *p* < 0.01.

**Table 3:** Regression models predicting youth pain and mental health during the COVID-19 pandemic.

	*β*	*B*	SE	*T*	*R* ^2^	95% CI	*p*
Model 1: Youth depression and Neuroticism							
*Block 1*					0.45		<0.01
Gender	0.03	1.14	3.52	0.32		−5.88 to 8.15	0.75
Age	0.15	1.24	0.76	1.64		−0.27 to 2.76	0.11
Pre-COVID depression	0.62 **	0.72	0.10	6.64		0.51 to 0.92	<0.01
*Block 2*					0.54		<0.01
COVID impact	0.13	0.18	0.12	1.48		−0.06 to 0.42	0.14
Neuroticism	0.31 **	0.85	0.25	3.43		0.36 to 1.34	<0.01
*Block 3*					0.55		<0.01
COVID impact * Neuroticism	0.10	0.02	0.02	1.22		−0.01 to 0.06	0.23
**Model 2: Youth depression and Extroversion**							
*Block 2*					0.52		
COVID impact	0.16	0.21	0.12	1.71		−0.04 to 0.46	0.09
Extroversion	−0.24 **	−0.65	0.24	−2.74		−1.13 to −0.18	<0.01
*Block 3*					0.54		
COVID impact * Extroversion	−0.14	−0.03	0.02	−1.75		−0.05 to 0.004	0.09
**Model 3: Youth depression and Conscientiousness**							
*Block 2*					0.54		
COVID impact	0.09	0.13	0.12	1.04		−0.12 to 0.37	0.30
Conscientiousness	−0.28 **	−0.83	0.25	−3.35		−1.32 to −0.34	<0.01
*Block 3*					0.54		
COVID impact * Conscientiousness	−0.02	−0.01	0.02	−0.20		−0.05 to 0.04	0.85
	***β***	***B***	**SE**	***T***	***R* ^2^**	**95% CI**	***p***
**Model 4: Youth anxiety and Neuroticism**							
*Block 1*					0.40		<0.01
Gender	0.08	2.77	3.33	0.83		−3.87 to 9.41	0.41
Age	0.18	1.27	0.68	1.86		−0.09 to 2.64	0.08
Pre-COVID anxiety	0.56 **	0.38	0.07	5.72		0.25 to 0.51	<0.01
*Block 2*					0.64		<0.01
COVID impact	0.20 *	0.22	0.09	2.42		0.04 to 0.40	0.019
Neuroticism	0.52 **	1.19	0.20	6.08		0.80 to 1.58	<0.01
*Block 3*					0.65		<0.01
COVID impact * Neuroticism	0.12	0.02	0.01	1.53		−0.01 to 0.05	0.13
**Model 5: Youth anxiety and Extroversion**							
*Block 2*					0.52		<0.01
COVID impact	0.24 *	0.26	0.10	2.53		0.06 to 0.47	0.014
Extroversion	−0.28 **	−0.62	0.20	−3.15		−1.01 to −0.23	<0.01
*Block 3*					0.54		<0.01
COVID impact * Extroversion	−0.14	−0.02	0.02	−1.69		−0.04 to 0.004	0.10
**Model 6: Youth anxiety and Conscientiousness**							
*Block 2*					0.49		<0.01
COVID impact	0.17	0.18	0.11	1.70		−0.03 to 0.40	0.09
Conscientiousness	−0.22 *	−0.51	0.22	−2.29		−0.95 to −0.07	0.03
*Block 3*					0.53		<0.01
COVID impact * Conscientiousness	−0.19 *	−0.05	0.02	−2.17		−0.09 to −0.003	0.034
	***β***	***B***	**SE**	***T***	***R* ^2^**	**95% CI**	***p***
**Model 7: Youth Pain Interference and Neuroticism**							
*Block 1*					0.25		<0.01
Gender	−0.003	−0.06	1.99	−0.03		−4.02 to 3.89	0.97
Age	0.08	0.35	0.43	0.82		−0.50 to 1.20	0.41
Pre-COVID pain interference	0.48 **	0.49	0.11	4.60		0.28 to 0.70	<0.01
*Block 2*					0.33		<0.01
COVID impact	0.27 *	0.19	0.07	2.59		0.04 to 0.33	0.01
Neuroticism	0.19	0.26	0.14	1.80		−0.03 to 0.55	0.08
*Block 3*					0.34		<0.01
COVID impact * Neuroticism	−0.10	−0.01	0.01	−0.97		−0.03 to 0.01	0.34
**Model 8: Youth Pain Interference and Extroversion**							
*Block 2*					0.35		<0.01
COVID impact	0.30 **	0.20	0.07	2.82		0.06 to 0.35	<0.01
Extroversion	−0.21 *	−0.29	0.14	−2.15		−0.57 to −0.02	0.04
*Block 3*					0.35		<0.01
COVID impact * Extroversion	0.10	0.01	0.01	1.00		−0.01 to 0.03	0.32
**Model 9: Youth Pain Interference and Conscientiousness**							
*Block 2*					0.34		<0.01
COVID impact	0.24 *	0.17	0.07	2.28		0.02 to 0.31	0.03
Conscientiousness	−0.19	−0.28	0.15	−1.93		−0.57 to 0.01	0.06
*Block 3*					0.35		<0.01
COVID impact * Conscientiousness	0.06						0.52

** *p* < 0.01, * *p* < 0.05. Note: Gender coded as 0 = female. Block 1 not reported multiple times for each outcome as results are identical prior to including block 2. Sample size: *n* = 80 for Models 1–3; *n* = 72 for Models 4–6; *n* = 81 for Models 7–9.

**Table 4:** Regression models predicting parent mental health during the COVID-19 pandemic.

	*β*	*B*	SE	*T*	*R* ^2^	95% CI	*p*
Model 1: Parent depression and Neuroticism							
*Block 1*					0.59		<0.01
Gender	−0.12	−1.30	0.77	−1.68		−2.83 to 0.23	0.10
Pre-COVID depression	0.77 **	0.98	0.09	10.95		0.80 to 1.16	<0.01
*Block 2*					0.61		<0.01
COVID impact	0.06	0.02	0.02	0.89		−0.03 to 0.07	0.37
Neuroticism	0.15 *	0.10	0.05	2.14		0.01 to 0.20	0.04
*Block 3*					0.62		<0.01
COVID impact * Neuroticism	0.11	0.01	0.003	1.61		−0.001 to 0.01	0.11
**Model 2: Parent depression and Extroversion**							
*Block 2*					0.63		
COVID impact	0.06	0.02	0.02	0.88		−0.03 to 0.06	0.38
Extroversion	−0.20 **	−0.13	0.05	−2.88		−0.22 to −0.04	<0.01
*Block 3*					0.64		
COVID impact * Extroversion	−0.12	−0.01	0.004	−1.83		−0.01 to 0.001	0.07
**Model 3: Parent depression and Conscientiousness**							
*Block 2*					0.59		
COVID impact	0.06	0.02	0.02	0.77		−0.03 to 0.07	0.44
Conscientiousness	−0.03	−0.03	0.06	−0.43		−0.14 to 0.09	0.67
*Block 3*					0.60		
COVID impact * Conscientiousness	−0.11	−0.01	0.004	−1.56		−0.02 to 0.002	0.12
**Model 4: Parent anxiety and Neuroticism**							
*Block 1*					0.53		
Gender	−0.11	−1.20	0.81	−1.48		−2.80 to 0.41	0.14
Pre-COVID anxiety	0.73 **	0.78	0.08	9.73		0.62 to 0.94	<0.01
*Block 2*					0.63		
COVID impact	0.18 *	0.06	0.02	2.60		0.01 to 0.10	0.01
Neuroticism	0.29 **	0.19	0.05	3.97		0.10 to 0.29	<0.01
*Block 3*					0.65		
COVID impact * Neuroticism	0.16 *	0.01	0.003	2.34		0.001 to 0.01	0.02
**Model 5: Parent anxiety and Extroversion**							
*Block 2*					0.64		
COVID impact	0.16 *	0.05	0.02	2.45		0.01 to 0.09	0.02
Extroversion	−0.30 **	−0.20	0.04	−4.60		−0.28 to −0.11	<0.01
*Block 3*					0.68		
COVID impact * Extroversion	−0.20 **	−0.01	0.003	−3.23		−0.02 to −0.004	<0.01
**Model 6: Parent anxiety and Conscientiousness**							
*Block 2*					0.58		
COVID impact	0.16 *	0.05	0.02	2.16		0.004 to 0.10	0.03
Conscientiousness	−0.16 *	−0.13	0.06	−2.25		−0.24 to −0.12	0.03
*Block 3*					0.60		
COVID impact * Conscientiousness	−0.15 *	−0.01	0.004	−2.10		−0.02 to 0	0.04

** *p* < 0.01, * *p* < 0.05. Note: gender coded as 0 = female. Block 1 not reported multiple times for each outcome as results are identical prior to including block 2. Sample size: *n* = 88 for Models 1–6.

## Supplementary Materials

The following supporting information can be downloaded at: https://www.mdpi.com/article/10.3390/children9050745/s1, Table S1: COVID-19-related personal impact questionnaire.
