# Supplementary material for: The Impact of the COVID-19 Pandemic on Youth with Chronic Pain and Their Parents: A Longitudinal Examination of Who Are Most at Risk
Source: Children (Basel). 2022 May 19;9(5):745. doi: 10.3390/children9050745 (PMC9139609; doi:10.3390/children9050745)
Supplement: Supplementary file 1 [file children-09-00745-s001.zip › children-1715702-supplementary.pdf]

Table S1. COVID Impact Questionnaire Means and Standard Deviations.

Likert Scale Questions

**Parent Questionnaire**		**Child Questionnaire**	
**Item**	**Mean (SD)**	**Item**	**Mean (SD)**
1. Did you or your family experience financial hardships?	1.75 (1.05)	1. Did you or your family experience financial (money) hardships?	1.73 (0.92)
2. Did you fear for your own or your family’s health or safety?	2.27 (1.01)	2. Did you fear for your own or your family's health or safety?	2.26 (1.08)
3. Did your children fear for their own or your family’s health or safety?	2.21 (1.02)	3. Did your parents fear for their own or your family’s health or safety?	2.33 (1.04)
4. To what extent was your life disrupted by the COVID-19 pandemic?	3.37 (1.15)	4. To what extent was your life disrupted by the COVID pandemic?	3.65 (1.04)
5. Did you have difficulty getting food or water?	1.21 (0.57)	5. Did you or your family have difficulty getting food or water?	1.06 (0.29)
6. Did you or your family experience a shortage of essential goods (e.g., toilet paper, cleaning supplies)?	1.50 (0.86)	6. Did you or your family experience a shortage of essential goods (e.g., toilet paper, cleaning supplies)?	1.31 (0.60)
7. Did you or your family have difficulty getting medicine?	1.23 (0.60)	7. Did you or your family have difficulty getting medicine?	1.21 (0.65)
8. Did you or your family have difficulty getting health care when you/they needed it?	1.64 (0.91)	8. Did you or your family have difficulty getting health care when you/they needed it?	1.36 (0.76)
9. Did you or your family have difficulty getting mental health care when you/they needed it?	1.53 (0.88)	9. Did you or your family have difficulty getting mental health care when you/they needed it?	1.64 (1.08)
10. Did your children fight, argue, or complain more than usual (if your child doesn’t have a sibling please select ‘not at all’)?	1.88 (1.08)	10. Did you and your siblings fight, argue, or complain more than usual (if you don’t have a sibling select ‘not at all’)?	2.19 (1.10)
11. Did you and your partner/spouse fight or argue more than usual or experience more conflict? (if you don’t have a partner/spouse, please select “not at all”).	1.62 (0.97)	11. Did your parents fight or argue more than usual or experience more conflict?	1.68 (1.02)

Likert Scale Questions (continued)

**Parent Questionnaire**		**Child Questionnaire**	
**Item**	**Mean (SD)**	**Item**	**Mean (SD)**
12. Did you and/or your partner/spouse fight or argue more than usual with your children or experience more conflict with them?	1.79 (1.02)	12. Did you and your parents fight or argue more than usual or experience more conflict?	1.90 (1.00)
13. Have you or your family felt “cramped”, or closed in, due to the physical distancing or quarantine?	2.37 (1.09)	13. Have you or your family felt “cramped”, or closed in, due to the physical distancing or quarantine?	2.42 (1.05)
14. Were you less able to see or interact with your friends?	3.59 (1.15)	14. Were you less able to see or interact with your friends?	3.69 (1.22)
15. Were you less physically active?	2.69 (1.28)	15. Were you less able to see your boyfriend or girlfriend? (if you don’t	1.58 (1.19)

Parent Questionnaire Item	Mean (SD)	Child Questionnaire Item	Mean (SD)
		have a boyfriend or girlfriend, please select “not at all”).	
16. Did you have difficulties associated with finding childcare?	1.32 (0.88)	16. Were you less physically active?	2.87 (1.40)
17. Did you use substances (e.g., alcohol, cannabis, or other substances) more than usual (i.e., more than the amounts you consumed before the COVID-19 pandemic)?	1.49 (0.72)	17. Did you have to stop your hobbies or interests?	2.27 (1.22)
18. On average, how much time did you spend each day reading/watching COVID-19 media (news, articles, social media)?	2.67 (1.08)	18. Did you use substances (e.g. alcohol, cannabis, or other substances) more than usual (i.e. more than the amounts you consumed before the COVID-19 pandemic?)	1.35 (0.86)
19. Were you in mandated quarantine or self-isolation (i.e., as forced by public health authorities for a specified period of time: staying within the home, no physical contact with individuals who live outside the household, maintaining 2 meters/6 feet of distance between you and people who do not live in your household).” If you were not mandated by public health to quarantine or self-isolate, please select “0 days”.	1.36 (0.91)	19. On average, how much time did you spend each day reading/watching COVID-19 media (news, articles, social media)?	1.92 (1.06)

Likert Scale Questions (continued)

Parent Questionnaire Item	Mean (SD)	Child Questionnaire Item	Mean (SD)
20. Did you practice “physical distancing”? (i.e., staying within the home, no physical contact with individuals who live outside the household, maintaining 2 meters/6 feet of distance between you and people who do not live in your household). If so, for how long?	4.04 (1.25)	20. Were you in mandated quarantine or self-isolation (i.e., as forced by public health authorities for a specified period of time: staying within the home, no physical contact with individuals who live outside the household, maintaining 2 meters/6 feet of distance between you and people who do not live in your household). If you were not mandated by public health to quarantine or self-isolate, please select “0 days”.	1.72 (1.34)
		21. Did you practice “physical distancing”? (i.e., staying within the home, no physical contact with individuals who live outside the household, maintaining 2 meters/6 feet of distance between you and people who do not live in your household). If so, for how long?	3.89 (1.41)

Frequency Questions

Parent Questionnaire Item	Yes/No (%)	Child Questionnaire Item	Yes/No (%)
21. Because of the COVID-19 pandemic, were you or your partner/spouse (or in the case of divorce/separation, your children’s other parent) ever required to be physically separated from	Yes = 8 (10.3)<br>No = 70 (89.7)	22. Did one of your parents lose their job, whether temporarily or permanently?	Yes = 17 (23.6)<br>No = 55 (76.4)

your children?	

Frequency Questions (continued)			
**Parent Questionnaire**		**Child Questionnaire**	
**Item**	**Yes/No (%)**	**Item**	**Yes/No (%)**
22. Did you or your partner/spouse have to apply for government financial aid?	Yes = 28 (35.9) No = 50 (64.1)	23. Did you lose your job, whether temporarily or permanently?	Yes = 13 (18.1) No = 59 (81.9)
23. Did you or your partner/spouse have to apply for employment insurance (e.g., EI)?	Yes = 16 (20.8) No = 61 (79.2)	24. During the COVID pandemic, was anyone in your household required to leave your house to do their job?	Yes = 47 (65.3) No = 25 (34.7)
24. Did you or your partner/spouse lose your job, whether temporarily or permanently?	Yes = 27 (34.6) No = 51 (65.4)	25. Is your parent a front-line health care worker or do they work in a healthcare setting where patients with COVID are/were being treated?	Yes = 9 (12.5) No = 63 (87.5)
25. During the COVID pandemic, were you required to leave your house to do your job?	Yes = 35 (44.9) No = 43 (55.1)		
26. Are you or your partner/spouse (or in the case of divorce/separation, your children's other parent) a front-line health care worker or do you or your partner/spouse work in a healthcare setting where patients with COVID are/were being treated?	Yes = 16 (20.5) No = 62 (79.5)		

Other Frequency Questions			
**Parent Questionnaire**		**Child Questionnaire**	
**Item**	**Label**	**Item**	**Label**
27. During the COVID pandemic, which best describes your living situation?	Adequate space = 61 (78.2) Limited space = 12 (15.4) Cramped = 5 (6.4)	26. During the COVID pandemic, which best describes your living situation?	Adequate space = 55 (76.4) Limited space = 14 (19.4) Cramped = 3 (4.2)

Abbreviation: SD, standard deviation.
